# The Royal Navy Medical Service

**Published:** 1911-09-16

**Authors:** 


### SPECIAL ARTICLES.

### THE ROYAL NAVY MEDICAL SERVICE.

The attitude of at least one of our contemporaries in abstaining from comment upon the Admiralty circular letter until the official regulations embodying the changes are issued does not commend itself to us. The senior service, when giving effect to changes which the Lords Commissioners have approved, is wont to carry out in the spirit as well as in the letter the various alterations that have received approval before they have been made public. Medical men contemplating a career in the Navy may rely upon the conditions in the future being wholly those which are set forth with much clearness in the circular letter [No. 24].

The Admiralty is desirous not only to meet the wishes of the many able men now serving afloat, but the whole spirit of the *communiqué* shows a desire to make the service what it should be, to attract to it men of standing and attainment who may look forward to a career of deep interest with the prospect of high rank and honours.

The Lords Commissioners of the Admiralty make definite statements as to the changes in the organisation and conditions of service in the Medical Branch of the Royal Navy, and state that these will take effect as from July 1 last. The first-mentioned alteration is the important change in the titles of the heads of the department. In future the Inspector-General and the Deputy Inspector-General of Hospitals and Fleets will be Surgeon-General and Deputy Surgeon-General Royal Navy, while "the medical officer of the senior flagship of a fleet is to be recognised as the principal medical officer of that fleet"; furthermore, he will be appointed on the staff of the Commander-in-Chief and wear an aiguillette. These last-mentioned privileges have long been withheld from the members of the medical profession serving in the Royal Navy. While the Flag Captain, the Flag Lieutenant, and the Secretary (a post always held by a paymaster) are attached to the staff and so decorated, the Fleet Surgeon has had no such privilege. But furthermore, the P.M.O. will now report periodically to the Admiralty on the general care and state of the sick, the sanitation of the ships of the fleet, the transport of the wounded in time of war, and such-like matters. Reports of surgeons serving on the various ships of the fleet will in future be referred to the P.M.O. for his opinion before they are sent in, the same arrangement taking place in naval establishments where there are naval hospitals and stationary ships.

The question of pay and emoluments is so arranged as to give general satisfaction, and places the naval surgeon on a very satisfactory footing as compared with other officers in the service of the Crown. The actual pay up to the rank of Fleet Surgeon remains the same as formerly, but instead of an officer having to wait four years or more for an increment, in the future he will receive an increase of pay every two years; for example, a Staff Surgeon on promotion received £365 per annum, and after four years in commission a further four shillings a day was added. At this rate of pay he had to remain until promoted to Fleet Surgeon at £492, a varying period according to the exigencies of the service. In future the difference between the pay of the two ranks will be so divided as to give an increment practically every two years. Thus, although there is no actual increase in the pay of a Staff or Fleet Surgeon on attaining that rank, the receipts of his office will be considerably augmented while passing from one rank to another. In this respect he will be in a far better position than officers in the Army, who frequently have to wait for seven and more years before a rise in pay is received. On attaining the rank of Deputy Surgeon-General the pay is £55 higher than the corresponding rank under the old regulations, while the staff at the Admiralty, the Director-General, Deputy and Assistant Directors-General all receive augmented pay. The flag allowance of the past is abolished, but service pay, which varies from 2s. 6d. to 7s. 6d. a day, now applies to a larger number of officers, and more than makes up for the pay formerly given when serving on a flagship.

The foreshadowed arrangements for post-graduate teaching and study leave are likely to prove as efficient as the Army Medical School at Millbank, which is mentioned in the Admiralty letter as one of the educational establishments that the Navy School will be in touch with. The great difference between these two schools is in the fact that the Army has its own hospital, to which the school is attached, while the Navy will be dependent upon the civil hospitals for clinical teaching and material for the pathological laboratories, etc. Otherwise, the Navy School will be equally well supplied with teachers from within the service, while the new laboratories which are to be constructed will rival those that now exist in any educational establishment in London. There is, however, one point to which attention may be drawn: the new laboratories, it is stated, will be all within the Royal Naval College at Greenwich. This is all very well as regards bacteriology, hygiene, etc., but a pathological laboratory, to render full efficiency and to be of full use to students, should be in close contiguity to the post-mortem rooms of a hospital and in close relationship to hospital wards. The Dreadnought Hospital mentioned as one of the units in the scheme stands within the grounds of the Royal Naval College, and it would be possible to erect the new pathological laboratories in close proximity to this hospital; if these laboratories are constructed in the college they will be a considerable distance from the hospital, and so lose a great part of their usefulness. So much of the school as will be carried on outside of the hospitals will be officered by the Medical Department of the Royal Navy. It is announced that the Professors of Bacteriology, Pathology, and Hygiene are to be selected from the Fleet Surgeons' list. Excellent work in this direction has already been done at Haslar, and we may anticipate that at any rate Bacteriology will be offered to Fleet Surgeon P. W. Bassett Smith; his name appeared in the last Honours for a C.B., and he has had a distinguished career. He was one of the first officers in the Royal Navy to realise the importance of naval surgeons being equipped with a knowledge of tropical medicine, and on being seconded for study took out the course of the London School of Tropical Medicine in 1899, subsequently taking the D.T.M. and H. of Cambridge in 1905, and gaining the Crags Research Prize of the Tropical School the following year. He has done much good work at Haslar. Fleet Surgeon W. W. Pryn, who has charge of surgeons on their entering the service, is another who may be expected at Greenwich. The Dreadnought Hospital and its staff will supply the greater part of the clinical instruction, and there is a possibility that some senior members of the Naval Medical Service may be added either to the hospital staff or to the staff of the London School of Clinical Medicine, which is attached to the Dreadnought Hospital. All surgeons on entering the service will in future be posted for a six months' course of instruction. The first two months will be spent at Greenwich, and the remainder of the period at Haslar as at present. Other officers of all ranks will be seconded from time to time for study, and senior men will be afforded an opportunity to specialise, and, though attached at Greenwich, to seek instruction in any recognised centre of medical education. Special arrangements will be made for a certain number of officers to attend the London School of Tropical Medicine for the three months' course.

The establishment of this school at Greenwich will not do away with the existing school at Haslar and the facilities for instruction at other naval hospitals. At Haslar, Chatham, and Plymouth systematic courses of six weeks' instruction will be arranged and facilities will be afforded to medical officers in their commands to attend them if they desire to do so.

The arrangements generally should enable those officers who are keen on their profession to keep themselves as well abreast of the advances in modern surgery and hygiene as the medical officers in any other branch of his Majesty's service. The circular letter goes on to recount various alterations and improvements in the conditions of the sick berth staff, the nursing service, and the reserve of naval medical officers.

